# Inactivation of Sirt1 in mouse livers protects against endotoxemic liver injury by acetylating and activating NF-*κ*B

**DOI:** 10.1038/cddis.2016.270

**Published:** 2016-10-06

**Authors:** Xiaolan Cui, Qian Chen, Zhen Dong, Longmei Xu, Tianfei Lu, Dawei Li, Jiangjun Zhang, Ming Zhang, Qiang Xia

**Affiliations:** 1 Department of Transplantation and Hepatic Surgery, Ren Ji Hospital, School of Medicine, Shanghai Jiao Tong University, Shanghai, China; 2 Department of Geriatric Cardiology, Chinese PLA General Hospital, Beijing, China; 3 Transplantation Center of the Affiliated Hospital of Qingdao University, Qingdao, Shandong, China; 4 The Central Laboratory of Ren Ji Hospital, School of Medicine, Shanghai Jiao Tong University, Shanghai, China

## Abstract

Sirtuin 1 (Sirt1) is a deacetylase that regulates many cellular processes in the liver, and so far its role in endotoxemic liver injury is elusive. So we conditionally inactivate Sirt1 in murine hepatocytes to determine its role in d-galactosamine (GalN)/lipopolysaccharide (LPS)-induced liver damage, which is a well-established experimental model mimicking septic liver injury and fulminant hepatitis. Ablation of Sirt1 shows remarkable protection against GalN/LPS-induced liver injury, which is a result of enhanced NF-*κ*B response because knockdown of RelA/p65 negates the protective effect of Sirt1 knockout. Mechanistically, NF-*κ*B p65 is maintained in a hyperacetylated, DNA-binding competent state in tumor necrosis factor-*α* (TNF-*α*)-challenged albumin-Cre^+^ (AlbCre^+^) hepatocytes. Transfection of hepatocytes with a recombinant acetylated p65 expression construct replicates the protection afforded by Sirt1 knockout. Transfection of AlbCre^+^ hepatocytes with a recombinant wild-type Sirt1 construct, rather than a deacetylase-defective one, compromises NF-*κ*B activation and resensitizes hepatocytes to TNF-*α*-induced apoptosis. Taken together, our results demonstrate that Sirt1 deacetylates p65 and compromises NF-*κ*B activity in hepatocytes when confronted with LPS/TNF-*α* stimulation, leading to increased susceptibility to endotoxemic injury. These findings identify a possible protein effector to maneuver the hepatic NF-*κ*B signaling pathway under inflammatory circumstances and a feasible way to increase hepatocellular resistance to endotoxin/TNF-*α* toxicity.

The development of liver dysfunction and failure is an important cause of morbidity and mortality in patients with Gram-negative sepsis and endotoxemia, especially in liver transplantation recipients.^1, 2^ It is widely accepted that the development of septic liver injury involves increased susceptibility of the liver to inflammation and oxidative stress.

The liver is the central metabolic organ as well as an important immune organ involved in inflammatory processes. In stress and injury, there is the generation of a hypermetabolic state and enhanced hepatic immunological activities.^3^ These functional changes are tightly regulated by gene expressions as well as post-translational modifications such as acetylation.

Sirtuin 1 (Sirt1) is a nuclear nicotinamide adenine dinucleotide (NAD+)-dependent protein deacetylase that functions as a key metabolic/energy sensor and regulates the transcriptional networks that control hepatic metabolism.^4, 5^ Even though the mechanisms of endotoxemic liver injury have been a subject of intensive investigations during the last two decades, the role of the ‘multifunctional' Sirt1 in the complex scenario of septic liver injury and whether Sirt1 is an ideal target for potential therapeutic interventions still remain poorly clarified.

Sirt1 is generally regarded as an important regulator that protects cells against stress and apoptosis. By regulating various targets, Sirt1 has roles in protein and cellular homeostasis during stress, thereby protecting cells from oxidative and genotoxic damage. ^6^ However, in the setting of endotoxemic liver injury, the role of Sirt1 is controversial. Both Sirt1 activators^7, 8^ and inhibitors^9^ have been reported to protect against endotoxin-induced hepatotoxicity, indicating a complex role of Sirt1 in septic liver failure.

In the present study, we generate hepatocyte-specific Sirt1 knockout mice to explore the role of Sirt1 in d-galactosamine (GalN)/lipopolysaccharide (LPS)-induced liver damage, which is a well-established experimental model mimicking septic liver injury and fulminant hepatitis. To our surprise, like other key proteins, Sirt1 turns out to have more than one face. Our results demonstrate that Sirt1 inactivation leads to hepatic protection both *in vivo* and *in vitro*, suggesting that Sirt1 can be ‘detrimental', and actually undermines the innate mechanisms that protect the liver in the setting of septic injury.

## Results

### Pharmacological inhibition or genetic inactivation of Sirt1 protects against GalN/LPS-induced liver injury

**Figure 1:**
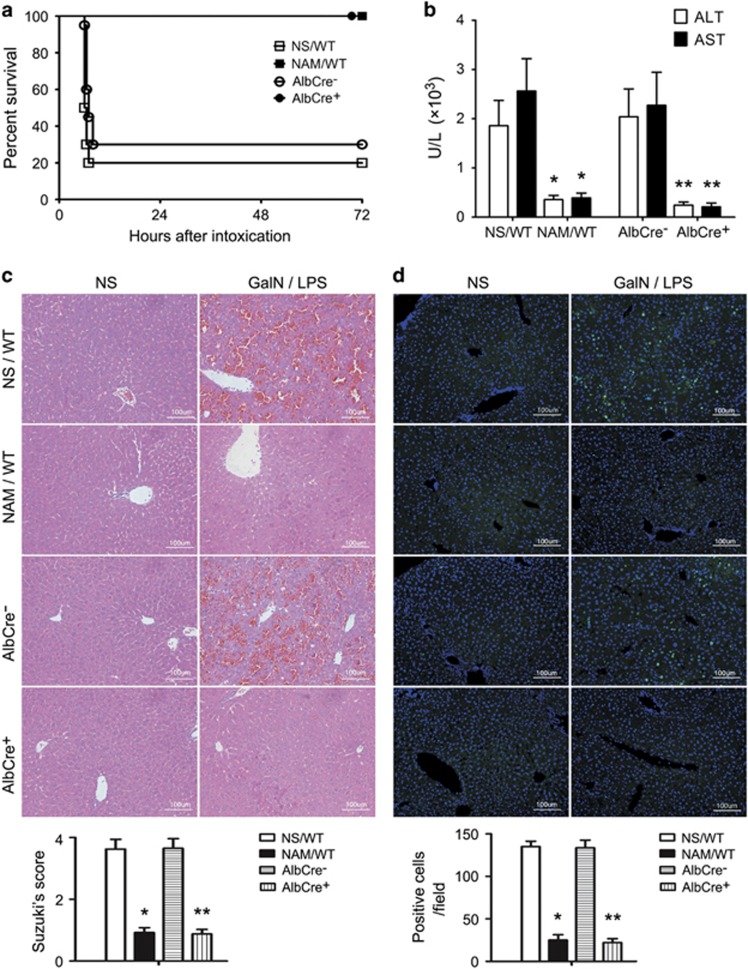
Sirt1 inhibition or inactivation protects against GalN/LPS-induced liver injury. Wild-type mice were subjected to oral administration of NAM (400 mg/kg) at 1 h before intraperitoneal injection of GalN (300 mg/kg)/LPS (20 *μ*g/kg). Control mice received normal saline (NS) as a vehicle before GalN/LPS. AlbCre^+^Sirt1^loxp/loxp^ mice (Sirt1^−/−^) and their Cre^−^ littermates (Sirt1^+/+^) received no previous treatment before GalN/LPS intoxication. (**a**) Survival of mice after intoxication (*n*=20 per group). NAM treatment led to a significant survival advantage by Kaplan–Meier analysis (log-rank test, *P*<0.05 between NAM and NS groups). Hepatocyte-specific (AlbCre^+^) inactivation of Sirt1 also led to a significant survival advantage (*P*<0.05 between AlbCre^+^ mice and AlbCre^-^ littermates). (**b**) Serum ALT and AST concentrations at 6 h after intoxication were shown (*n*=4 per group). *P<0.05 *versus* NS-treated controls; **P<0.05 *versus* Cre^−^ littermates. (**c**) Representative hematoxylin- and eosin-stained sections from normal livers and post-intoxication livers collected at 6 h (original magnification, × 200). GalN/LPS-induced hemorrhagic necrosis was absent in NAM-treated wild-type (WT) mice or AlbCre^+^ Sirt1 knockout mice. (**d**) Representative sections from terminal deoxynucleotidyl transferase dUTP nick end labeling assay (original magnification, × 200)

Sirt1 functions to produce nicotinamide (NAM), overload of which thus inhibits Sirt1 activity. In the present study, NAM was administered orally to mice at 1 h before intraperitoneal injection of GalN/LPS (300 mg/20 μg/kg). At 6 h after the intoxication, mice were killed to obtain blood and liver samples. Separate groups of mice were subjected to survival experiments. As shown in Figure 1a, without NAM treatment, death of mice occurred at 6 h and 80% mice died within 2 h thereafter. NAM treatment showed remarkable protection and led to 100% survival at 7 days in all mice. Blood aminotransferase (alanine aminotransferase (ALT) and aspartate aminotransferase (AST)) tests and pathological findings were consistent and shown in Figures 1b–d. NAM given at 1 h after GalN/LPS also showed protective effect and led to ~70% survival in intoxicated mice (data not shown). To explore the role of Sirt1 in this setting, we employed Sirt1 knockout mice. Albumin-Cre (AlbCre)-induced genetic disruption of Sirt1 in hepatocytes also led to significant protection against GalN/LPS-induced liver failure (Figures 1a–d).

### Inactivation of Sirt1 in hepatocyte promotes and prolongs NF-*κ*B-binding activity by maintaining p65 in an acetylated, DNA-binding competent state

**Figure 2:**
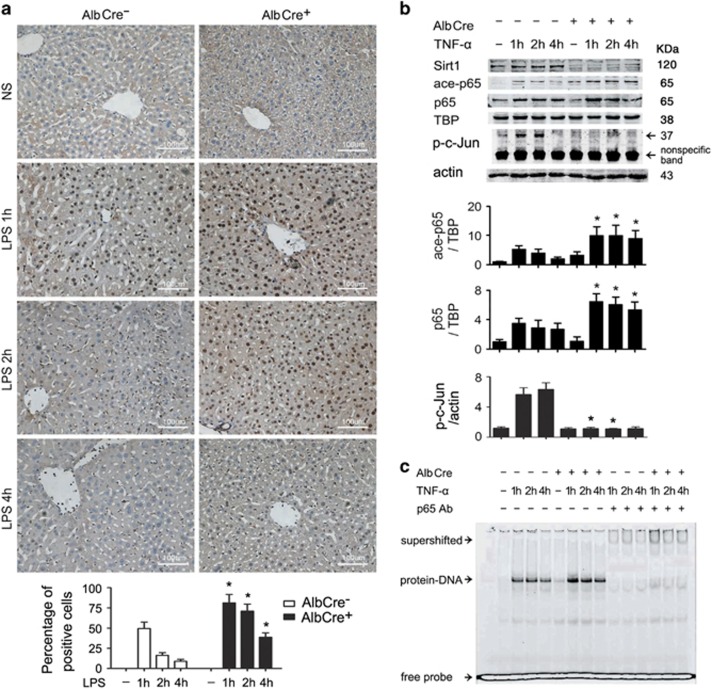
NF-*κ*B p65 is maintained in an acetylated, DNA-binding competent state in LPS/TNF-*α*-challenged AlbCre^+^ livers/hepatocytes. (**a**) Both AlbCre^+^ Sirt1 knockout mice and AlbCre^−^ littermates were injected intraperitoneally with LPS (100 *μ*g/kg), followed by collection of liver samples at the indicated time points. Mice that were injected with NS served as controls. Hepatic samples were fixed in formalin, embedded in paraffin and processed for immunohistochemical localization of acetylated NF-*κ*B p65. Representative photographs are shown (original magnification, × 200). Similar results were obtained in six independent experiments. (**b**) Primary hepatocytes were isolated from both AlbCre^–^ and AlbCre^+^ mice. TNF-*α* was added to cultured hepatocytes to obtain a final concentration of 20 ng/ml in culture medium. At 1, 2 and 4 h after the addition of TNF-*α*, hepatocytes were collected and nuclear expression of Sirt1, acetylated and total p65 were detected by western blot and co-detection of TATA-binding protein (TBP) was performed to assess equal loading. Target protein bands were quantified and normalized to TBP. JNK kinase activity was measured by the detection of phosphorylation of a c-Jun substrate, and co-detection of *β*-actin was performed to assess equal loading. Note the nonspecific background band below p-c-Jun target band was found in all samples, which also indicates equal loading. The mean value obtained from AlbCre^−^ cells without treatment was arbitrarily defined as 1. There were four samples from different individuals at every time point in each group, and the data were expressed as the mean±S.D. *P<0.05 *versus* Cre^−^ counterparts. (**c**) Same amount of nuclear extracts as used in immunoblotting assays were subjected to gel mobility shift assays using a DyLight 680-labeled NF-*κ*B consensus probe. NF-*κ*B p65 antibody was added to the reaction to generate supershifts. The protein–DNA and supershifted complexes were indicated, respectively

Mice were injected intraperitoneally with LPS, followed by harvest of liver samples at different time points. Liver samples were then stained immunohistochemically with an antibody against acetylated NF-*κ*B p65. As shown in Figure 2a, LPS treatment caused nuclear accumulation of acetylated p65 in AlbCre^−^ controls. However, the NF-*κ*B response was stronger and lasted much longer in AlbCre^+^ livers.

Tumor necrosis factor-*α* (TNF-*α*) is the predominant mediator that induces hepatocyte apoptosis and liver injury in LPS-induced injury. To look into the mechanisms behind the enhanced NF-*κ*B response in AlbCre^+^ livers, primary hepatocytes were isolated from both AlbCre^−^ and AlbCre^+^ mice. TNF-*α* was added to cultured hepatocytes, which were then collected, and nuclear Sirt1 and acetylated/total p65 were detected by western blot. As shown in Figure 2b, TNF-*α* treatment did not change Sirt1 protein levels in hepatocytes. Consistent with the *in vivo* results, AlbCre^+^ hepatocytes showed stronger nuclear p65 accumulation and much lower JNK activity after TNF-*α* treatment, which was in line with p65 acetylation. NF-*κ*B-binding activity to a consensus promoter sequence was determined by electrophoretic mobility shift assay (EMSA) analysis using the same amount of nuclear extracts as described in the western blot studies. As shown in Figure 2c, Sirt1 knockout created a much more competent NF-*κ*B-binding capacity in TNF-*α*-challenged hepatocytes. Consistently, Sirt1 knockout leads to the overexpression of NF-*κ*B downstream genes after TNF-*α* treatment (Supplementary Figure 1).

### Knockdown of RelA/p65 counteracts the protective effect of Sirt1 inactivation

**Figure 3:**
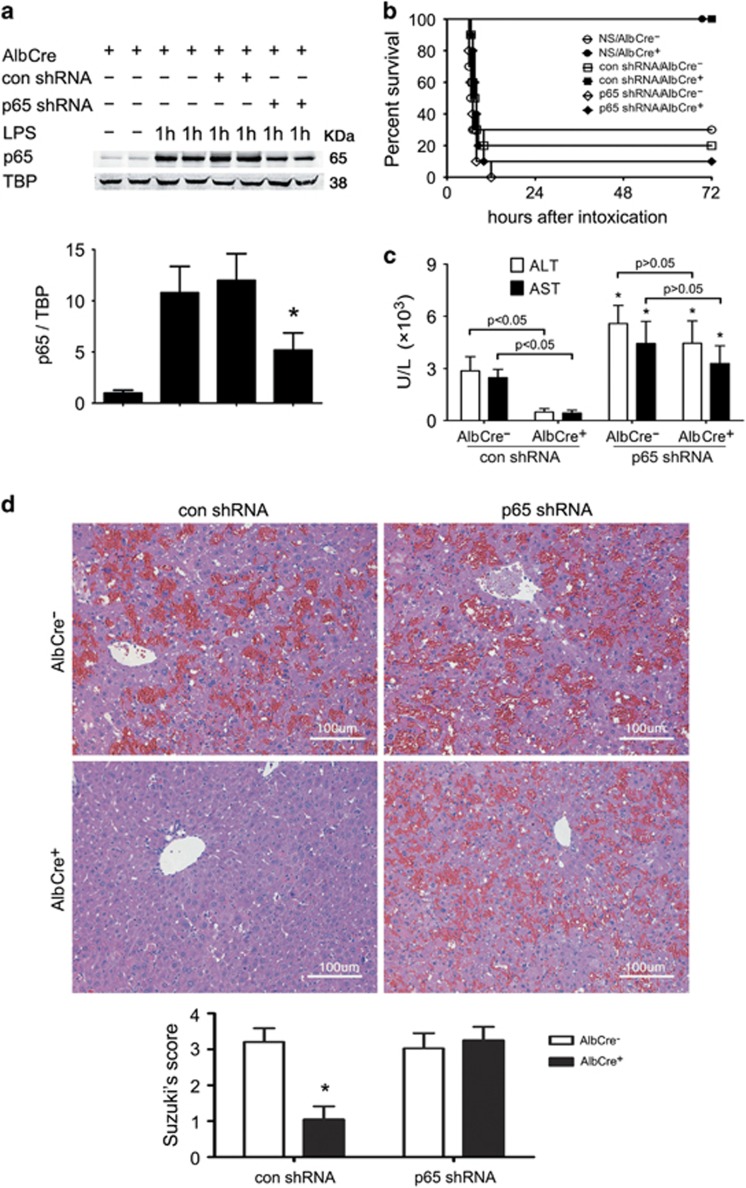
Knockdown of RelA/p65 compromises the hepatoprotection in AlbCre^+^ mice. NF-*κ*B p65 shRNA plasmid or a control (con) plasmid was administered to AlbCre^+^ mice or wild-type (WT) littermates by a hydrodynamic-based gene transfer technique via rapid injection of a large volume of DNA solution through the dorsal vein of penis. (**a**) At 48 h after the administration of the plasmid, mice were injected intraperitoneally with LPS (100 *μ*g/kg). At 1 h after LPS treatment, liver samples were collected and subjected to immunoblotting analysis using an antibody against NF-*κ*B p65. The mean value obtained from mice without treatment was arbitrarily defined as 1. There were four mice in each group and data were expressed as the mean±S.D. *P<0.05 *versus* con shRNA-treated group. (**b**) At 48 h after the administration of the plasmid, mice were injected intraperitoneally with GalN/LPS (300 mg/20 *μ*g/kg). Survival of mice after the intoxication was shown (*n*=10 per group). Compared with con shRNA plasmid, p65 shRNA resulted in a significant survival disadvantage in AlbCre^+^ mice by Kaplan–Meier analysis (log-rank test, *P*<0.05 between con shRNA and p65 shRNA groups). (**c**) Serum ALT and AST concentrations at 6 h after intoxication were shown (*n*=4 per group). *P<0.05 *versus* con shRNA-treated syngeneic mice. Statistical significance and insignificance were shown. (**d**) Representative hematoxylin- and eosin-stained sections from livers collected at 6 h after the intoxication (original magnification, × 200). In AlbCre^+^ mice, GalN/LPS-induced hemorrhagic necrosis was absent in con shRNA-treated but not in p65 shRNA-treated mice

To dissect the role of the augmented NF-*κ*B response in the hepatoprotection observed in Sirt1-deficient mice, NF-*κ*B p65 short hairpin RNA (shRNA) plasmid was administered by a hydrodynamic-based gene transfer technique via rapid injection of a large volume of DNA solution through the dorsal vein of penis, as described previously.^10^ The procedure effectively inhibited p65 expression in the liver (Figure 3a). At 48 h after the administration of the plasmid, mice were subjected to GalN/LPS intoxication. As shown in Figure 3b, p65 shRNA plasmid negated the protective effect of Sirt1 knockout, leading to over 90% death in AlbCre^+^ mice. The result was reinforced by observations from aminotransferase tests and pathological examinations (Figures 3c and d).

**Figure 4:**
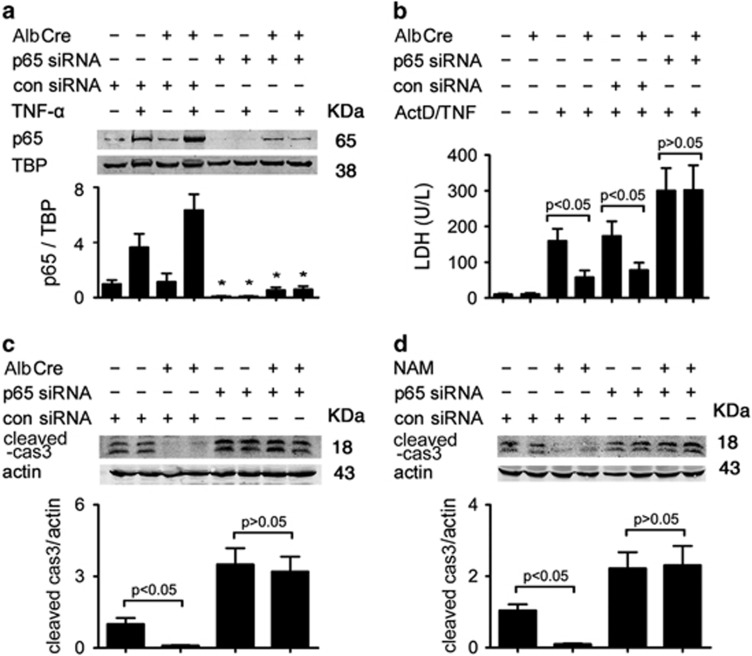
Knockdown of RelA/p65 compromises the resistance of AlbCre^+^ hepatocytes against ActD/TNF-*α*. Primary hepatocytes were transfected with siRNA duplexes targeting mouse NF-*κ*B p65 at a final concentration of 20 nM for 48 h. (**a**) TNF-*α* (20 ng/ml) was added to cultured hepatocytes. Two hours later, hepatocytes were collected and nuclear p65 was detected. The mean value obtained from AlbCre^−^ cells in the control siRNA group without TNF-*α* treatment was arbitrarily defined as 1. *P<0.05 *versus* control siRNA-treated counterparts. (**b**) The hepatocytes were subjected to intoxication by ActD/TNF-*α* (20 ng/20 ng/ml) for 24 h. The supernatant was then collected and LDH concentration was measured colorimetrically. (**c**) Cells were collected at 6 h after ActD/TNF-*α* treatment and whole-cell proteins were obtained to detect cleaved caspase 3. The mean value obtained from AlbCre^−^ cells in the control siRNA group was arbitrarily defined as 1. (**d**) Primary hepatocytes isolated from wild-type mice were treated with NAM (10 mM) at 2 h before ActD/TNF-*α*. At 6 h after the intoxication, cells were collected to detect cleaved caspase 3. The mean value obtained from the control siRNA group without NAM treatment was arbitrarily defined as 1. There were four samples from four different individuals in each group and data were expressed as mean±S.D. Statistical significance and insignificance were indicated

Cultured primary hepatocytes were also subjected to knockdown treatment by small interfering RNA (siRNA) duplex targeting mouse NF-*κ*B p65. The treatment inhibited p65 expression in hepatocytes, and the inhibition was more significant than *in vivo* results (Figure 4a). Freshly isolated mouse hepatocytes were essentially insensitive to TNF-*α* cytotoxicity. However, TNF-*α* induced a concentration-dependent cell death in hepatocytes that had been pretreated with the transcriptional inhibitors actinomycin D (ActD).^11^ Consistent with *in vivo* results, AlbCre^+^ hepatocytes were much less sensitive to ActD/TNF-*α*-induced cytotoxicity than AlbCre^−^ cells, as evidenced by much less lactate dehydrogenase (LDH) release and apoptosis induction (Figures 4b and c). Pretreatment with p65 siRNA resensitized these cells to ActD/TNF-*α* and eliminated the difference between AlbCre^+^ and AlbCre^−^ hepatocytes. We also pretreated wild-type hepatocytes with NAM, which elicited protection against ActD/TNF-*α*-induced cytotoxicity. Knockdown of NF-*κ*B p65 negated the effect of NAM, indicating that as a Sirt1 inhibitor, the effect of NAM was also based on NF-*κ*B activation (Figure 4d).

To further dissect the role of acetylated p65 in Sirt1 knockout-induced hepatoprotection, we constructed the adenovirus producing mouse p65, in which lysine 310 was replaced by a glutamine (K310Q) to mimic constitutive acetylation. Primary hepatocytes were infected for 1 h at an multiplicity of infection (MOI) of 10. After 48 h, cells were challenged with TNF-*α*. Adenovirus-mediated gene transfer successfully improved the expression of acetylated p65 in hepatocytes, which greatly reduced ActD/TNF-induced apoptosis and eliminated the difference between AlbCre^+^ and AlbCre^−^ hepatocytes (Supplementary Figure 2).

### Transfection of AlbCre^+^ hepatocytes with a recombinant wild-type Sirt1 expression construct, rather than a deacetylase-defective Sirt1 mutant, compromises NF-*κ*B activation and resensitizes hepatocytes to TNF-induced apoptosis

**Figure 5:**
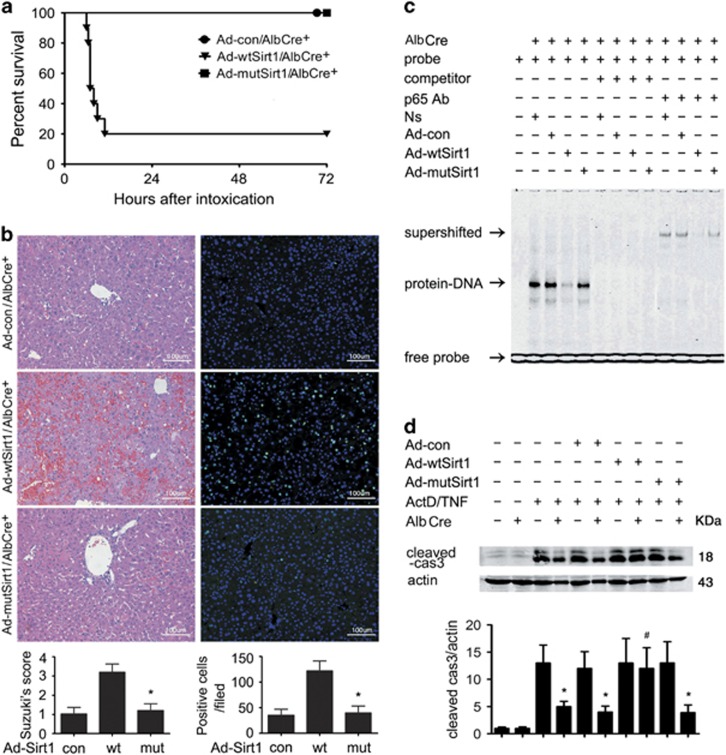
Overexpression of a wild-type, rather than deacetylase-defective Sirt1 construct, compromises NF-*κ*B activation and resensitizes AlbCre^+^ hepatocytes to TNF-induced apoptosis. AlbCre^+^ mice were intravenously injected with 2 × 10^9^ infective units of adenoviruses containing either full-length mouse Sirt1 (Ad-wtSirt1) or a deacetylase-defective Sirt1 mutant (Ad-mutSirt1). (**a**) Ten days later, these mice were subjected to GalN/LPS (300 mg/20 *μ*g/kg) injection and survival after intoxication was shown (*n*=10 per group). Compared with Ad-con and Ad-mutSirt1 groups, overexpression of wild-type Sirt1 (Ad-wtSirt1) led to a significant survival disadvantage by Kaplan–Meier analysis (log-rank test, *P*<0.05). (**b**) Representative hematoxylin- and eosin-stained, and terminal deoxynucleotidyl transferase dUTP nick end labeling sections from post-intoxication livers collected at 6 h (original magnification, × 200). (**c**) Primary AlbCre^+^ hepatocytes were transfected with Ad-wtSirt1, Ad-mutSirt1 or control virus (Ad-con). At 48 h, TNF-*α* was added to culture medium (20 ng/ml). Two hours later, cells were collected and nuclear extract was subjected to electrophoretic mobility shift assay using a DyLight 680-labeled consensus NF-*κ*B probe. No nuclear extract was added to the first lane. Supershift assay showed that NF-*κ*B band was shifted because of the formation of bigger complex after addition of anti-NF-*κ*B p65 antibody. Hundredfold molar excess of unlabeled probe was used as competitor. (**d**) Primary hepatocytes from both AlbCre^+^ and AlbCre^−^ mice were transfected with adenoviruses at 48 h before ActD/TNF-*α* (20 ng/20 ng/ml) challenge. Cells were collected at 6 h after the intoxication to detect cleaved caspase 3. The mean value obtained from Cre^−^ cells without treatment was arbitrarily defined as 1. There were four samples from four different individuals in each group and data were expressed as mean±S.D. *P<0.05 *versus* Cre^−^ counterparts. ^#^*P*>0.05 *versus* Cre^−^ cells transfected with Ad-wtSirt1

Although Sirt1 is a well-confirmed deacetylase, Sirt1 can function through physical protein–protein interaction independent of the deacetylase activity. To clarify whether the inhibition of its deacetylase activity was responsible for the protective effect observed in the present study, we constructed adenoviruses producing full-length mouse Sirt1 (Ad-wtSirt1) or a deacetylase-defective (H355Y) Sirt1 mutant (Ad-mutSirt1). Overexpression of these recombinant constructs in the liver was accomplished via tail vein injection of adenoviruses (2 × 10^9^ plaque-forming unit) at 10 days before GalN/LPS intoxication. Transfection of AlbCre^+^ livers with wild-type Sirt1 resensitized these mice to GalN/LPS-induced toxicity, but overexpression of the dominant-negative mutant Sirt1 did not make a difference from the Ad-con group (Figures 5a and b). Mechanistically, transduction in primary AlbCre^+^ hepatocytes with the wild-type Sirt1, rather than the mutant construct, attenuated NF-*κ*B-binding activity (Figure 5c), which was inversely correlated with apoptosis induction (Figure 5d).

### Sirt1 Knockout also promotes p53 acetylation in TNF-*α*-challenged hepatocytes

**Figure 6:**
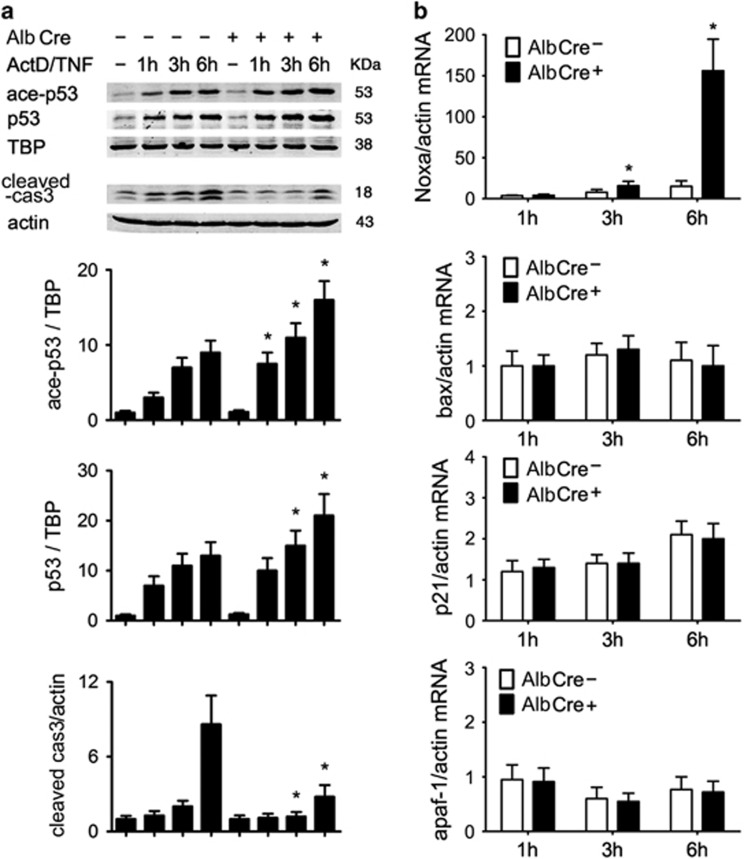
Sirt1 knockout leads to p53 hyperacetylation and a selective induction of Noxa. Primary hepatocytes were isolated from both AlbCre^−^ and AlbCre^+^ mice, and subjected to ActD/TNF-*α* (20 ng/20 ng/ml) challenge. (**a**) At 1, 3 and 6 h after the addition of ActD/TNF-*α*, cells were collected, and nuclear expressions of acetylated and total p53 were detected. The mean value obtained from AlbCre^−^ cells without treatment was arbitrarily defined as 1. (**b**) At 1, 3 and 6 h after the addition of ActD/TNF-*α*, cells were collected and the expressions of Noxa, bax, p21 and apaf-1 were determined by quantitative real-time RT-PCR and were normalized to *β*-actin expression. Data were acquired from four independent experiments. All data were expressed as mean±S.D.; *P<0.05 *versus* Cre^−^ counterparts. No statistical difference was observed between AlbCre^−^ and AlbCre^+^ cells in the expressions of bax, p21 and apaf-1

p53 is another important transcription factor that induces apoptosis in cells exposed to noxious stresses by transcriptional activation of its target genes. To find out whether p53 and its target genes were influenced by Sirt1 knockout, primary hepatocytes were treated with ActD/TNF-*α*. At 1, 3 and 6 h after the intoxication, hepatocytes were collected and nuclear expression of both acetylated and total p53 were detected by western blot. As shown in Figure 6a, p53 was quickly induced and acetylated in AlbCre^+^ hepatocytes, indicating that Sirt1 was implicated in p53 regulatory pathway. Although it was reported that Sirt1 inactivation and p53 acetylation increased the sensitivity of cells in p53-dependent apoptotic response,^12^ in the present study AlbCre^+^ hepatocytes showed much less apoptosis induction. Real-time RT-PCR analysis was also performed to test whether p53 was transcriptionally more active in AlbCre^+^ hepatocytes. As shown in Figure 6b, the gene for the BH3-only protein Noxa was selectively induced in AlbCre^+^ hepatocytes, but expressions of other p53-dependent proapoptotic genes were unchanged. So, although Sirt1 inactivation promoted p53-dependent transcriptional activation, the hepatocytes were actually protected against TNF-*α*-induced apoptosis. These results indicate that p53-dependent apoptotic response does not dominate the events happening in Sirt1-deficient hepatocytes.

## Discussion

The results based on knockout mice indicate that hepatocyte Sirt1 is a detrimental rather than protective factor in the setting of endotoxemic liver injury. These findings are important, because unraveling the less common negative effect of Sirt1 may lead to a more comprehensive understanding of this generally accepted ‘beneficial gene'.

Although Sirt1 is a well-confirmed deacetylase and the catalytic activity is responsible for most of its biological effects, Sirt1 also functions through physical protein–protein interaction independent of the deacetylase activity under both stress and steady-state conditions.^13, 14, 15, 16^ To exclude a possible role of non-deacetylase Sirt1, we transduced Sirt1-deficient hepatocytes by adenoviruses with either wild-type mouse Sirt1 or a point mutant with impaired deacetylase activity (Sirt1 H355Y in which histidine at position 355 was replaced by tyrosine, corresponding to H363Y in human). Reintroduction of wild-type Sirt1, but not the mutant, resensitized AlbCre^+^ mice to GalN/LPS-induced liver injury, indicating that Sirt1 impaired the hepatocytes via its deacetylase activity.

Through its deacetylase activity, Sirt1 is able to either repress or activate the transcriptional activities of multiple targets, thereby regulating diverse metabolic and stress pathways. The earliest and the well-established Sirt1 substrates are RelA/p65 subunit of NF-*κ*B^17^ and the p53 tumor suppressor protein,^18^ both of which control the expression of gene products that affect key cellular processes determining cell survival or death. In the present study, we found that Sirt1 deacetylated both NF-*κ*B p65 and p53 in TNF-*α*-challenged hepatocytes. However, among all the p53 target genes tested, only Noxa was selectively induced in AlbCre^+^ hepatocytes. The result that the hyperacetylated p53 selectively promotes the expression of certain target genes but spares others suggests that there is a differentiated control on p53 functions by Sirt1, and also, unlike the DNA damage response, LPS/TNF-*α*-induced apoptosis occurs in a p53-independent manner and other factors than p53 dominate the events happening in AlbCre^+^ hepatocytes.

Hepatocyte resistance to TNF-*α*-induced apoptosis is dependent on the activation of NF-*κ*B and subsequent macromolecular synthesis. That hepatocytes lose the ability to effectively and appropriately activate the NF-*κ*B signaling pathway under TNF-*α* stimulation is a common reason that they fail to resist TNF-*α*-induced cell death.^19, 20^ In the present study, we did reveal NF-*κ*B to be the ‘special bodyguard' of Sirt1-deficient hepatocytes, in which hyperacetylated p65 led to augmented NF-*κ*B activity, prolonged NF-*κ*B response and finally resistance to LPS/TNF-*α*-dependent injury.

Acetylation of p65 is an important post-translational modification of NF-*κ*B and dynamic process that prevents reconnecting with the inhibitor of NF-*κ*B, and thus is critical for nuclear retention and transcription factor activity of NF-*κ*B. So, reversible acetylation of p65 serves as an important intranuclear molecular switch that controls the duration of the NF-*κ*B transcriptional activity.^21^ In this study, we found that p65 was hyperacetylated in AlbCre^+^ hepatocytes that featured stronger and longer NF-*κ*B response both *in vivo* and *in vitro*. These results indicate how this ‘molecular switch' is controlled in hepatocytes. Knockdown of p65 eliminated the difference between Sirt1-deficient and -sufficient hepatocytes, indicating that the augmented and prolonged NF-*κ*B response was responsible for the increased resistance to LPS/TNF-*α*-induced injury. It is worth notice that the context of LPS/TNF-*α* challenge is essential to Sirt1 knockout-induced hepatoprotection because LPS and TNF-*α* are the most common inducers of NF-*κ*B in the liver,^22^ whereas Sirt1 functions to deacetylate and deactivate it. Actually, in other liver injury models such as ischemia-reperfusion injury or acetaminophen liver damage, Sirt1 knockout failed to show the protective effects (Supplementary Figure 3), probably because of a lack of rapid nuclear NF-*κ*B accumulation in the first place.

Using non-small-cell lung cancer cells in their seminal study, Yeung *et al.*^17^ demonstrated that Sirt1 inhibited NF-*κ*B transactivation potential by directly deacetylating the RelA/p65 protein at lysine 310, and thus augmented apoptosis in response to TNF-*α*. Their results were reinforced by observations from other groups that demonstrated Sirt1 also suppressed inflammatory responses through the promotion of p65 deacetylation in macrophage cell lines.^23, 24^ In the present study, we found that the regulatory relationship between Sirt1 and NF-*κ*B also existed in hepatocytes. Our findings are both biologically and clinically significant, because these results provide novel information regarding the etiology of endotoxemic liver injury.

The liver is more vulnerable to septic injury than most organs. The consensus of opinion is that the liver collects blood from gastrointestinal tract and thus is continuously exposed to a lot of endotoxin, which makes the liver a frontline/filter organ and susceptible to endotoxemic injury. Our results may introduce an important but less-acquainted player at the molecular level in this setting, that is, the actively functioning Sirt1 in the liver that compromises hepatic NF-*κ*B activity. As we know, whole-body nutrient homeostasis, such as glucose, lipid and cholesterol, is critically regulated by the liver, in which Sirt1 is an energy status sensor and has many important roles in tuning liver metabolism to nutrient availability and energy balance.^4, 25, 26, 27, 28, 29^ Correspondingly, Sirt1 is constitutively expressed in the liver.^18^ In this study, we have shown that after TNF-*α* treatment, Sirt1 level in hepatocytes remains hardly changed, suggesting that even under the stressful condition the metabolic machinery driven by Sirt1 is not inhibited. On the other hand, Sirt1 transfers the acetyl group of lysines in a protein substrate such as NF-*κ*B p65, to the ADP-ribose moiety of NAD+ to produce a deacetylated protein, 2′-*O*-acetyl-ADP ribose and NAM. So, Sirt1 is a NAD+-dependent deacetylase and the activity of Sirt1 is closely controlled by different environmental cues that change the cellular NAD+ availability. It is worth notice that the liver is the tissue with the strongest expression of enzymes that produce NAD+.^30^ Thus, the liver features the high-level, actively functioning Sirt1, together with the high availability of its substrate. Our findings thus explain, at least partially, why the liver is vulnerable to septic injury. It is interesting and worth notice that the lung, another organ with high-level Sirt1 expression,^21^ is also highly susceptible to LPS-induced acute injury.

Taken together, our results demonstrate that Sirt1 deacetylates NF-*κ*B p65 and thus compromises NF-*κ*B activity in hepatocytes under LPS/TNF-*α* stimulation, leading to increased susceptibility to endotoxemic injury. These findings identify a possible protein effector to maneuver hepatic NF-*κ*B signaling pathway under inflammatory circumstances and a feasible way to increase hepatocellular resistance to endotoxin/TNF-*α* toxicity.

## Materials and Methods

### Mice

The Cre/loxP recombination system was used to generate Sirt1 knockout mice, as described previously.^31^ AlbCre transgene mice (stock number: 003574) and Sirt1 floxed mice (008041) were both from the Jackson Lab (Bar Harbor, ME, USA). The mating strategy, genotyping and the confirmation of target gene excision were described in the Supplementary Methods section. Littermates lacking the Cre recombinase (Cre^−^) were used as controls. Wild-type C57BL/6 mice were purchased from Shanghai SLAC Co. Ltd (Shanghai, China).

Male mice, 8–14 weeks of age and weighing 20–28 g, were used in the present study. All mice received humane care and were housed in a pathogen-free facility and handled in accordance with the institutional guidelines of Shanghai Jiao Tong University, School of Medicine, and the ‘Guide for the Care and Use of Laboratory Animals' prepared by the National Academy of Sciences and published by the National Institutes of Health (NIH publication 86-23 revised 1985). All the procedures described were approved by the Animal Use and Care Committee of Shanghai Jiao Tong University, School of Medicine (approval number: SYKX-2012-0031).

### Drugs and experimental design

All substances that were not otherwise specified were purchased from Sigma-Aldrich (St Louis, MO, USA). Acute liver failure was induced by intraperitoneal injection of GalN (300 mg/kg) plus LPS (20 *μ*g/kg, from *Escherichia coli* serotype 055:B5). Some mice received LPS (100 *μ*g/kg) only, followed by the harvest of liver samples and immunohistochemical detection of RelA/p65. NAM (400 mg/kg) was suspended in normal saline (NS) and administered for once by gavage at 1 h before GalN/LPS intoxication. Animals in control groups received NS orally, followed by GalN/LPS injection. Animals were killed at different time points after GalN/LPS administration by exsanguination to obtain blood and liver samples for further analyses.

### *In vivo* knockdown of NF-*κ*B p65 by hydrodynamics-based shRNA injection

NF-*κ*B p65 shRNA plasmid (sc-29411-SH) and a control shRNA plasmid (sc-108060) were purchased from Santa Cruz Biotechnology (Dallas, TX, USA). To inhibit p65 expression in the liver, we used a hydrodynamics-based transfection method to deliver naked shRNAs to the mouse liver, as described previously.^32^ In brief, after anesthesia with sevoflurane, shRNA plasmid (10 *μ*g in 2 ml of Ringer's solution) was injected into the dorsal veins of penis within 5 s. At 48 h after shRNA injection, mice were treated with GalN/LPS or LPS alone.

### Construction and purification of adenoviruses containing recombinant p65 or Sirt1 expression constructs

We amplified the consensus coding sequence of mouse RelA/p65 by PCR, in which lysine 310 was replaced by a glutamine (K310Q) to mimic constitutive acetylation. After it was cloned into the entry vector (pMD18-T), the sequence was confirmed. The expression construct was then recombined into the Gateway-based pAd-CMV/V5-DEST vector (Invitrogen by Thermo Fisher Scientific, Waltham, MA USA) and was termed Ad-ace-p65. We also constructed adenoviruses producing full-length mouse Sirt1 (Ad-wtSirt1), a deacetylase-defective (H355Y) Sirt1 mutant (Ad-mutSirt1) and a control virus that contained only EGFP but did not include the expression sequence (Ad-con). The procedures for the production and purification of recombinant adenovirus were in accordance with the manufacturer's instruction manual. Amplification of recombinant adenovirus was performed using HEK 293A cells. For *in vivo* infection of adenoviruses, the mice were intravenously injected with a volume of 200 *μ*l containing 2 × 10^9^ infective units of viruses. The experiments were performed at 10 days after injection of the virus. In some experiments, primary hepatocytes were infected by adenoviruses for 1 h at an MOI of 10, and were then cultured in fresh medium for 48 h before used in further studies.

### Determination of lethality

Survival was observed after GalN/LPS intoxication as described previously.^33^ The number of survival mice was counted and recorded every half an hour from 4 to 12 h after the GalN/LPS injection, then every 12 h till the seventh day after intoxication. No deaths were observed after the third day, so mice that survived over 72 h were considered to survive indefinitely. If an animal was considered possibly morbid, the condition of the animal was monitored every 15 min. The presence of morbid symptoms was determined by an experienced observer with no prior information regarding the treatments and genetic background of the animals. Animals were considered morbid if they were severely immobile, hunched in posture, experiencing severe hypothermia and/or unresponsive to noise. After signs of morbidity were detected, death was considered unavoidable and the animal was killed under anesthesia with isoflurane inhalation. After that, a laparotomy was conducted and liver failure was confirmed by macroscopic and microscopic examination. Animals that survived to the seventh day were also killed under anesthesia, and the successful recovery of hepatic function was confirmed by serum ALT/AST analyses and macroscopic/microscopic examination.

### Biochemical analyses

Arterial blood was collected by direct puncture of arteriae aorta. Serum ALT and serum AST levels were measured with a standard clinical automatic analyzer (Dimension Xpand; Siemens Dade Behring, Munich, Germany).

### Liver histopathology, immunohistochemistry and terminal deoxynucleotidyl transferase dUTP nick end labeling assay

Hepatic samples were fixed in 10% neutral buffered formalin overnight, dehydrated, embedded in paraffin, sectioned and stained with hematoxylin and eosin.

The histological severity of liver injury was graded using Suzuki's criteria. In brief, sinusoidal congestion, hepatocyte necrosis and ballooning degeneration are graded from 0 to 4. No necrosis, congestion or centrilobular ballooning is given a score of 0, whereas severe congestion and ballooning degeneration as well as >60% lobular necrosis is given a value of 4. Some sections were processed for immunohistochemical localization of acetylated NF-*κ*B p65 (acetyl K310; ab52175, from Abcam, Cambridge, MA, USA), and were then visualized with diaminobenzadine and counterstained with hematoxylin. For histological analysis, sections were evaluated in a blinded manner by a pathologist. At least three fields per section were evaluated. Apoptotic cells were identified with an apoptosis detection kit (S7110, EMD Millipore Co., Merck KGaA, Darmstadt, Germany), as described previously.^31, 34^ Cells with nuclear-positive staining by fluorescent antibodies for DNA fragmentation were visualized directly by a fluorescence microscopy and counted (original magnification, × 200). At least three fields per section were examined.

### Mouse hepatocyte isolation and ActD/TNF-*α* intoxication

Mouse hepatocytes were isolated from male mice (8–12 weeks old), as described previously.^35^ In brief, hepatocytes were isolated by liver perfusion of collagenase I (Gibco, A1048301). The cells were purified by 90% Percoll (Sigma) density centrifugation. The viability of freshly isolated hepatocytes was >90% as confirmed by trypan blue exclusion. Isolated hepatocytes were suspended in culture medium and were allowed to attach in a gassed atmosphere (5% CO_2_) at 37 °C for 2 h. After the establishment of monolayers, the medium was removed and replaced with fresh medium containing TNF-*α* (20 ng/ml) or ActD/TNF-*α* (20 ng/20 ng/ml). Then, the hepatocytes were incubated for appropriate time periods, which were indicated in the figure legends. After incubation, the medium was collected for required biochemical assays and cells were collected to isolate proteins. In some experiments, NAM (10 mM) was added to the culture medium at 2 h before ActD/TNF treatment.

### RNA interference in primary hepatocytes

Double-stranded siRNA corresponding to homologous sequence of mouse RelA/p65 gene (sc-29411) or a nonspecific negative control (sc-37007) were purchased from Santa Cruz Biotechnology. RNA interference was performed as described previously.^36^ In brief, primary mouse hepatocytes in maintenance medium without serum and antibiotics were distributed onto 24-well or 60-mm culture plates and incubated for 3 h at 37 °C and 5% CO_2_ to allow cell adherence. Then, cells were transfected with siRNA duplexes at a final concentration of 20 nM in Opti-Mem (Life Technologies Co., Grand Island, NY, USA), using X-tremeGENE siRNA Transfection Reagent (Roche, Indianapolis, IN, USA) for 48 h, followed by TNF-*α* or ACTD/TNF-*α* treatment.

### Measurement of LDH release

To quantify cell death, the supernatant from cultured hepatocytes was collected and LDH concentration was measured colorimetrically using the CytoTox 96 nonradioactive cytotoxicity assay (Promega, Madison, WI, USA).

### Protein isolation and western blotting

Western blot analyses were conducted as described in a previous article.^33^ In brief, nuclear extracts were isolated from the livers or cultured hepatocytes, using NE-PER Nuclear and Cytoplasmic Extraction Reagents (Product Number 78833, Pierce Biotechnology, Rockford, lL, USA), supplemented with Complete Protease Inhibitor Cocktail Tablets (Roche). Membranes containing nuclear protein fractions were blocked with LI-COR blocking buffer and were then incubated with rabbit primary antibodies against Sirt1 (1:1000; #2028, Cell Signaling Technology, Danvers, MA, USA), NF-*κ*B p65 (1:2000, #4764, Cell Signaling Technology), acetyl-NF-*κ*B p65 K310 (1:1000, #3045, Cell Signaling Technology), p53 (1:1000, ab131442, Abcam) and acetyl-p53 K379 (1:1000, #2570, Cell Signaling Technology). After washing, membranes were incubated in Tris-buffered saline/0.05% Tween 20 buffer with IRDye800 secondary antibodies. The blot was visualized using an Odyssey infrared imaging system (LI-COR Biosciences, Lincoln, NE, USA). Samples were corrected for background and quantified using Odyssey software. All values were normalized to a loading control TATA-binding protein (1:2000; ab818, Abcam) and expressed as the fold increase relative to control.

For detection of cleaved caspase 3, solubilized whole-cell proteins were transferred to nitrocellulose membranes and were incubated with anti-Cleaved Caspase 3 (1:1000; #9664, Cell Signaling Technology). Blots were developed as described above and the expression levels were normalized to *β*-actin (1:2000; Santa Cruz Biotechnology).

### JNK assay

JNK kinase assays were performed using a commercial kit (#8794, Cell Signaling, Beverly, MA, USA), as previously described.^20^ JNK activity was measured by phosphorylation of a c-Jun substrate that was detected by immunoblotting with an antibody specific for c-Jun phosphorylated at serine 63. As a control for equivalent loading among protein samples, immunoblots for to *β*-actin were also performed (1:2000; Santa Cruz Biotechnology).

### Electrophoretic mobility shift assay

Protein–DNA interaction was detected using an Odyssey Infrared EMSA kit (LI-COR Biosciences), according to the manufacturer's protocols. In brief, nuclear extracts from hepatocytes were assembled with a DyLight 680-labeled double-stranded NF-*κ*B consensus oligonucleotide for EMSA (Takara Co., Dalian, China). The sense sequence was 5′-AGTTGAGGGGACTTTCCCAGGC-3′. The signal was detected and quantified using Odyssey infrared imaging system (LI-COR Biosciences). Hundredfold molar excess of unlabeled probe was used as competitor. Supershift assays were conducted using an anti-NF-*κ*B p65 antibody (ab7970, Abcam). Both assays were conducted to confirm the specificity of NF-*κ*B /DNA-binding activity.

### RT-PCR

Total RNA was extracted from hepatocytes using Trizol Reagent (Takara Co.) according to the manufacturer's instructions. Reverse transcription was performed using 1000 ng of total RNA in the first-strand cDNA synthesis reaction with PrimeScript RT reagent Kit (Takara). RT-PCR was performed using an ABI 7900 sequence detector (Invitrogen). RT-PCR was performed using SYBR Premix Ex Taq (Takara) and values were normalized to *β*-actin expression. Primer sequences were as follows: Noxa Forward: 5′-GGAAGTCGCAAAAGAGCAGGATG, and reverse: 5′-CTGCCGTAAATTCACTTTGTCTCC. Bax forward: 5′-AGGATGCGTCCACCAAGAAGCT-3′ and reverse: 5′-TCCGTGTCCACGTCAGCAATCA-3′. P21 forward: 5′-TCGCTGTCTTGCACTCTGGTGT-3′ and reverse: 5′-CCAATCTGCGCTTGGAGTGATAG-3′. Apaf-1 forward: 5′-CACGAGTTCGTGGCATATAGGC-3′ and reverse: 5′-GGAAATGGCTGTCGTCCAAGGA-3′. cIAP2 forward: 5′-GGACATTAGGAGTCTTCCCACAG-3′ and reverse: 5′-GAACACGATGGATACCTCTCGG-3′. A1/Bfl-1 forward: 5′-TCCACAAGAGCAGATTGCCCTG-3′ and reverse: 5′-GCCAGCCAGATTTGGGTTCAAAC-3′. iNOS forward: 5′-GAGACAGGGAAGTCTGAAGCAC-3′ and reverse: 5′-CCAGCAGTAGTTGCTCCTCTTC-3′. *β*-Actin forward: 5′-CATCCGTAAAGACCTCTATGCCAAC-3′ and reverse: 5′-ATGGAGCCACCGATCCACA-3′.

### Statistical analysis

All values were reported as the mean±S.D. Data were analyzed with a one-way analysis of variance with subsequent Student–Newman–Keul's test or Student's *t-*test where applicable. Statistical significance was set at *P*<0.05.

## Abbreviations

ActD: actinomycin D
Alb: Albumin
ALT: alanine aminotransferase
AST: aspartate aminotransferase
EMSA: electrophoretic mobility shift assay
GalN: d-galactosamine
LDH: lactate dehydrogenase
LPS: lipopolysaccharide
MOI: multiplicity of infection
NAD+: nicotinamide adenine dinucleotide
NAM: nicotinamide
NS: normal saline
shRNA: short hairpin RNA
siRNA: small interfering RNA duplex
Sirt1: Sirtuin 1
TNF-*α*: tumor necrosis factor-*α*
