# Supplementary material for: Inactivation of Sirt1 in mouse livers protects against endotoxemic liver injury by acetylating and activating NF-κB
Source: Cell Death Dis. 2016 Oct 6;7(10):e2403–. doi: 10.1038/cddis.2016.270 (PMC5133964; doi:10.1038/cddis.2016.270)
Supplement: Supplementary Figure Legends [file cddis2016270x2.docx]

**Supplementary Figure Legends**

**Supplementary Figure 1.Sirt1 knockout leads to overexpression of NF-κB downstream genes in TNF-α-challenged hepatocytes.**Primary hepatocytes were isolated from both AlbCre– and AlbCre+ mice and subjected to ActD/TNF-α (20 ng/20 ng/ml) challenge or vehicle control. At 1, 2, 4 h after the addition of ActD/TNF-α, cells were harvested and the expressions of NF-κB downstream genes cIAP2, A1/Bfl-1 and iNOS were determined by quantitative real-time RT-PCR and were normalized to β-actin expression. Data were acquired from 4 independent experiments. All data were expressed as mean ± SD; *P < 0.05 vs Cre- counterparts.

**Supplementary Figure 2.Overexpression of a recombinant acetylated p65 expression construct protects against ActD/TNF-induced injury and eliminates the difference between Sirt1-deficient and sufficient hepatocytes.** (A) Primary hepatocytes were transfected with Ad-ace-p65 or control virus (Ad-con) at an MOI of 10. At 48 hours, TNF-α was added to obtain a final concentration of 20 ng/ml in culture medium. 2 hours later, cells were harvested and nuclear extract was subjected to western blot analysis. The mean value obtained from Cre- cells in Ad-con group was arbitrarily defined as 1. *P < 0.05 vs Cre- cells in Ad-con group. (B) Primary hepatocytes were transfected with adenoviruses at 48 h before ActD/TNF-α (20 ng/20 ng/ml) challenge. Cells were harvested at 6 h after the intoxication and cleaved caspase 3 was detected. The mean value obtained from Cre- cells without treatment was arbitrarily defined as 1. There were four samples from four different individuals in each group and data were expressed as mean ± SD. Statistical significance and insignificance were indicated.

**Supplementary Figure 3Hepatocyte-specific inactivation of Sirt1 doesn’t protect against ischemia-reperfusion (IR) or acetaminophen (APAP)-induced liver injury.** Eight-week-old male Sirt1^-/-^ mice (AlbCre+) and wildtype littermates (AlbCre-) were subjected to 60 min ischemia followed by 6 hours of reperfusion (A) or intraperitoneal injection of APAP (300 mg/kg body weight) and blood samples were harvested at 6 h (B). Serum levels of ALT and AST were measured and shown. Data were presented as mean ± SD from six mice per group. #, P > 0.05 versus Cre- counterparts. (C) Representative images of HE stained liver sections collected at 6 h (original magnification, 200×).

**Supporting Methods**

**Mice** **and Genotyping.**The Cre/loxP recombination system was used to generate hepatocyte-specific Sirt1^-/-^ mice. Sirt1^loxP/loxP^ (stock number: 008041) and Albumin-Cre transgene mice (003574) were from the Jackson Laboratory (Bar Harbor, Maine USA). The mating strategy, genotyping and the confirmation of target gene excision were as described in a previous report([1](#_ENREF_1)). A PCR-based genotyping method was established by using genomic DNA isolated from tail biopsies to identify the wild-typeand floxed alleles of the Sirt1 gene using thefollowing primers: 5'-GGTTGACTTAGGTCTTGTCTG-3’; and 5'-CGTCCCTTGTAATGTTTCCC-3’, which identified the floxed allele as a 750-bp band and the wildtype allele as a 550-bp band. To confirm the knockout of the floxed exons, PCR-based genotyping was applied using DNA isolated from livers and the following primers: 5'-AGGCGGATTTCTGAGTTCGA-3’and5’-CGTCCCTTGTAATGTTTCCC-3’, which produced a 450-bp band in post cre excision alleles and a 900-bp band in wildtype alleles.Albumin-Cre transgene was routinely monitored by PCR using the following primers that produced a 478-bp product. Cre-F: 5’-AGGTGTAGAGAAGGCACTTAGC-3’ and Cre-R: 5’-CTAATCGCCATCTTCCAGCAGG-3’.

**Mouse Warm Hepatic IRI Model.**Liver partial warm IR was performed as described in our previous report([2](#_ENREF_2)). In brief, mice were anesthetized with isoflurane inhalation and were placed on a temperature-controlled heating table with a rectal thermometer probe attached to a thermal feedback controller (ALC-HTP Homeothermic System, Shanghai Alcott Biotech Co. Ltd, Jiading District, Shanghai, China) to maintain core temperature at 36°C. Then a microclip (Aesculap FT222T) was placed around the pedicle of the left lateral lobe to occlude both inflow and outflow for 60 minutes, after which the clip was removed to initiate the reperfusion. Mice were sacrificed at 6 h to obtain blood and liver samples for further analyses.

**APAP Treatment.**The mice were fasted overnight (16 hours) before APAP administration (from Sigma-Aldrich, St. Louis, MO, USA). APAP was dissolved in phosphate buffered saline and intraperitoneally administrated into mice at a dose of 300 mg/kg body weight. Animals were killed at 6 h after the intoxication by exsanguination, to obtain blood and liver samples for further analyses.

1. Li H, Rajendran GK, Liu N, Ware C, Rubin BP, Gu Y. SirT1 modulates the estrogen-insulin-like growth factor-1 signaling for postnatal development of mammary gland in mice. *Breast cancer research : BCR* 2007;**9**:R1.

2. Zhang J, Zhang M, Xia Q. A Novel Mouse Model of Liver Ischemic/Reperfusion Injury and its Differences to the Existing Model. *Journal of investigative surgery : the official journal of the Academy of Surgical Research* 2015;**28**:283-91.
