# Supplementary figures and images for: Inactivation of Sirt1 in mouse livers protects against endotoxemic liver injury by acetylating and activating NF-κB
Source: Cell Death Dis. 2016 Oct 6;7(10):e2403–. doi: 10.1038/cddis.2016.270 (PMC5133964; doi:10.1038/cddis.2016.270)

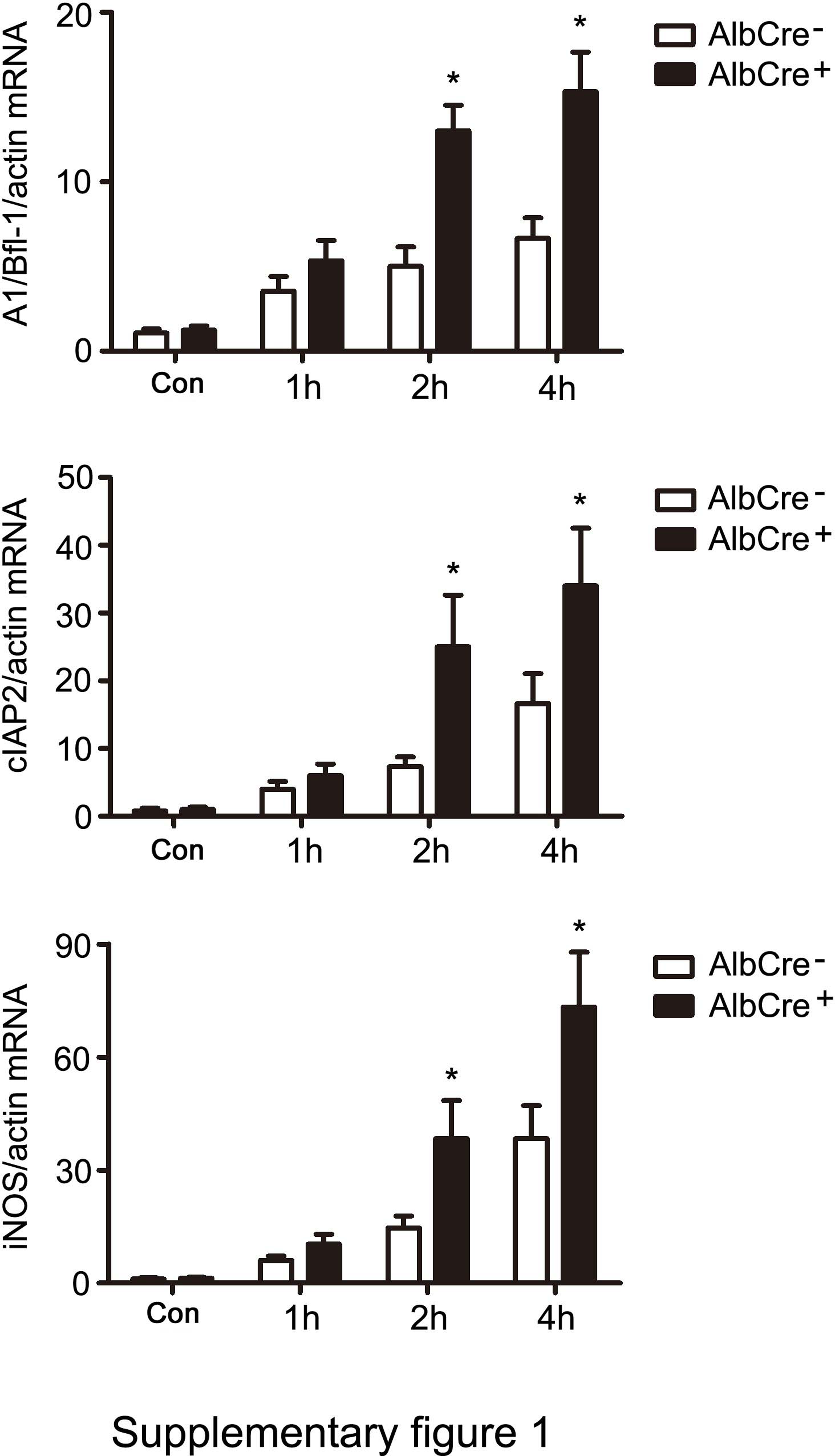

Supplement: Supplementary Figure 1 [file cddis2016270x3.tif]

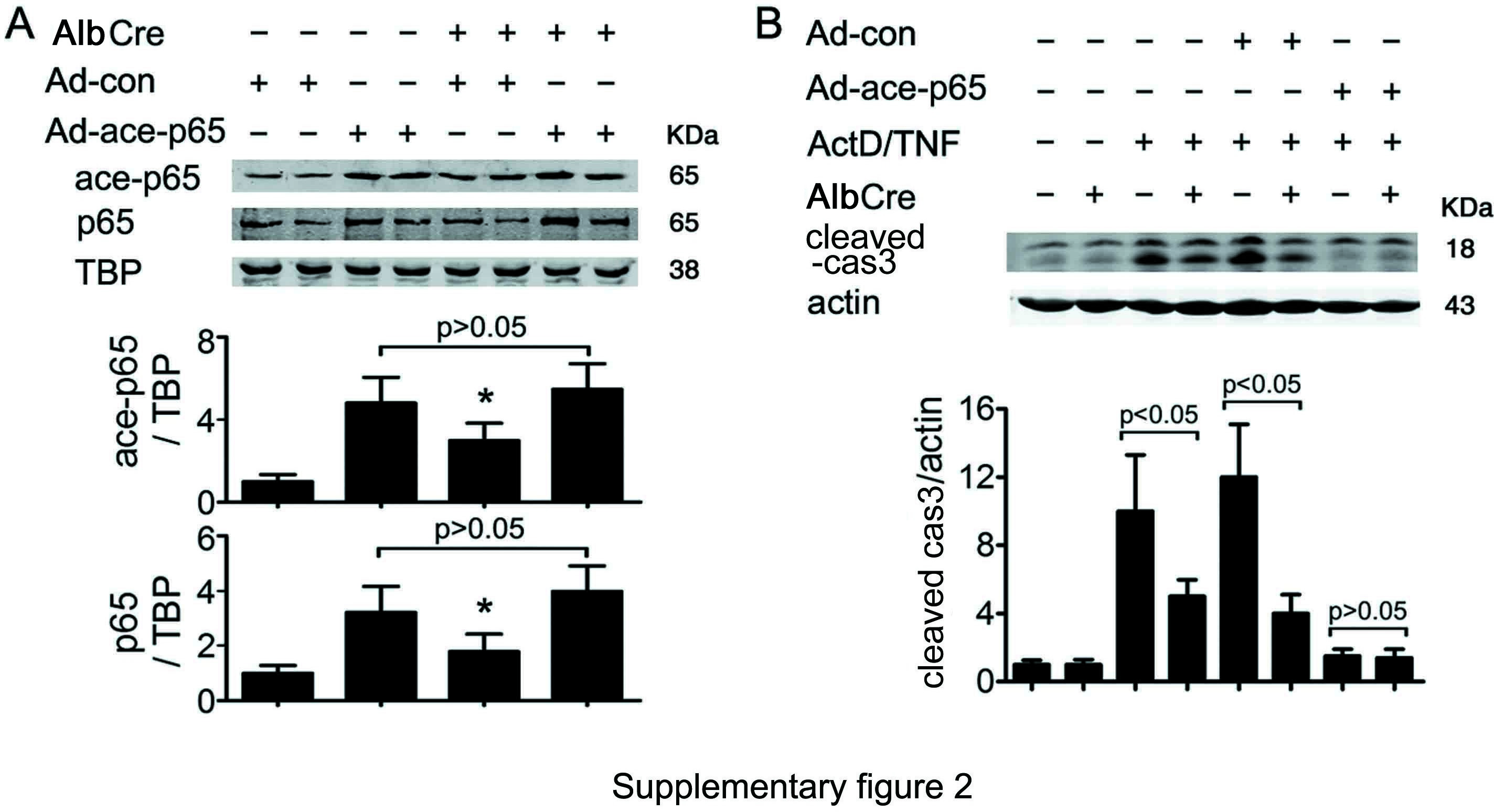

Supplement: Supplementary Figure 2 [file cddis2016270x4.tif]

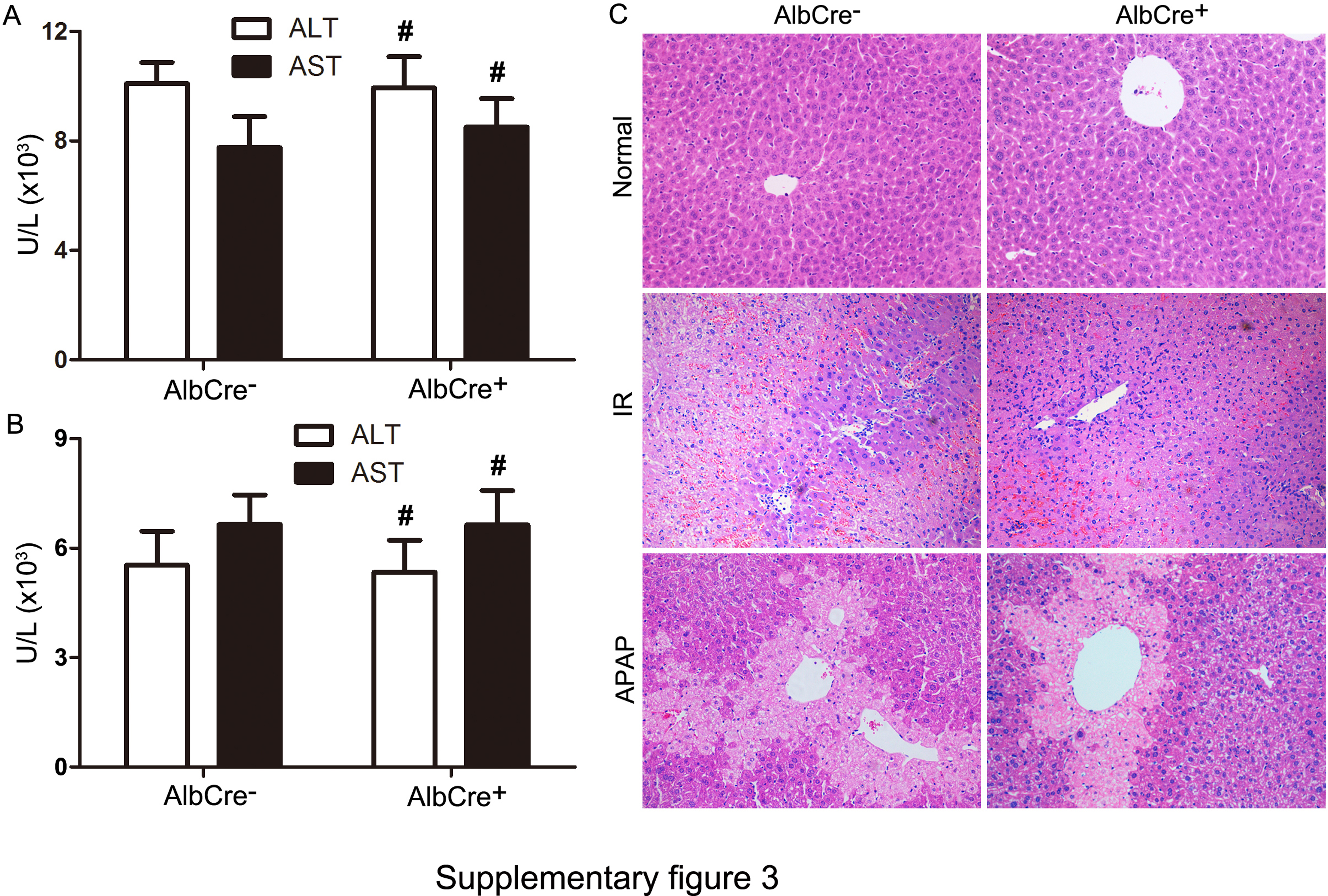

Supplement: Supplementary Figure 3 [file cddis2016270x5.tif]
